# Body condition as a descriptor of American alligator (*Alligator mississippiensis*) health status in the Greater Everglades, Florida, United States

**DOI:** 10.1371/journal.pone.0295357

**Published:** 2023-11-30

**Authors:** Sergio A. Balaguera-Reina, Laura A. Brandt, Nicole D. Hernandez, Brittany M. Mason, Christopher D. Smith, Frank J. Mazzotti

**Affiliations:** 1 Fort Lauderdale Research and Education Center, Institute of Food and Agricultural Science, University of Florida, Davie, Florida, United States of America; 2 U. S. Fish and Wildlife Service, Davie, Florida, United States of America; 3 Welleby Veterinary Hospital, Sunrise, Florida, United States of America; University of Bucharest, ROMANIA

## Abstract

Body condition is used as an indicator of the degree of body fat in an animal but evidence of its actual relationship with health diagnostics (e.g., blood parameters) is usually lacking across species. In American alligators (*Alligator mississippiensis*), body condition has been used as a performance metric within the Greater Everglades ecosystem to provide insight on hydrological and landscape changes on alligator populations. However, there is no clear evidence that spatial body condition changes relate to different health conditions (low food intake vs sickness) and whether this link can be made when relating body condition values with blood parameters. We assessed the relationship between alligator body condition and 36 hematological and biochemistry (blood) parameters in four areas across two physiographic regions (Everglades and Big Cypress) of the Greater Everglades (sample size = 120). We found very strong to weak evidence of linearity between 7 (Big Cypress) and 19 (Everglades) blood parameters and relative condition factor index, from which cholesterol (38%) and uric acid (41%) for the former and phosphorus (up to 52%) and cholesterol (up to 45%) for the latter (mean absolute error MAE = 0.18 each) were the predictors that individually explain most of the body condition variation. The best combination of blood parameters for the Everglades were cholesterol, phosphorus, osmolality, total protein, albumin, alpha 2, beta, and gamma globulins, and corticosterone accounting for 40% (37 ± 21%, MAE = 0.16) of the variation found in alligator body condition for this region. We found better predictability power in models when analyzed at smaller rather than larger scales showing a potential habitat effect on the body condition—blood parameters relationship. Overall, Everglades alligators in poorer condition are likely dehydrated or have an inadequate diet and the spatial differences found between physiographic regions suggest that these areas differ in prey availability/quality.

## Introduction

Body condition indices, the relationship between two morphometric variables (commonly weight and length) as proxy of physical fitness and health status (e.g., relative condition factors, Fulton’s K), are applied frequently in ecology, wildlife management, and conservation biology to assess individual and population level health as well as to understand how well animals are coping with their environment [1, 2]. It has been generally assumed to be related to energy stores (i.e., fat) [2, 3] and recently empirically tested to influence aspects of individual fitness such as growth [4], survival [5], and reproductive success [6]. However, there is currently a lack of research focused on understanding how body condition relates to health diagnostic metrics such as hematological and biochemistry (blood) parameters, specifically in reptiles. Research on juvenile American alligators (*Alligator mississippiensis*; hereafter alligator) [7] suggested some biochemistry parameters such as aspartate aminotransferase, creatine kinase, and globulin levels (commonly used in the diagnosis of liver/muscle injury or septicemia and dehydration or antigenic stimulation, respectively) [8] correlate to variations in body condition. This finding may imply that body condition can be related to health status in alligators. However, the small sample size and extent of that study limited further conclusions.

Alligators are endemic to the United States and are considered both keystone and flagship species [9]. In Florida, alligators are ecologically and economically important, making them of interest to local and federal land managers and wildlife managers [9]. Alligators are currently used as indicators of ecological responses to Everglades’ restoration to help guide restoration efforts because their body condition is closely tied to hydrology [9], making the species a perfect study subject to understand how health conditions relate to changes in habitat. Independent research efforts have been done in the Everglades in the last decades to understand body condition [10–12] and hematological and biochemistry parameters [13, 14]. However, there have been no published papers linking these two sets of variables to evaluate their relationships and the potential use of body condition as a composite metric of nutritional physiology, physical fitness, and health.

Hematological and biochemistry parameter values provide a minimally invasive way to evaluate wildlife health under a range of environmental conditions [15, 16]. They can be used to compare the health of different segments of a population (*e*.*g*., individuals inhabiting different interconnected areas) as well as to identify animals exposed to different stressors. On a broader scale, blood parameters are a standard tool that provides a benchmark for which to assess an individual’s health, and in clinical situations, make decisions on treatments [17, 18]. Several biochemistry parameters are related to nutritional status in wildlife populations. For example, elevated cholesterol, elevated triglycerides, and glucose variation may indicate that an animal has higher internal fat reserves. Variations to hydroxybutyrate values could indicate that an animal has been fasting and muscle digestion has been taking place [7, 19]. Corticosterone is related to fasting and poor body condition tied to environmental stressors (i.e., low rainfall) [20]. However, these parameters are prone to variation across populations, geographic location, and demographics [18], making it difficult to use them across wildlife studies unless the source of this variation is understood.

The physiological significance of body condition and its relationship with nutritional physiology, physical fitness, and overall health is warranted so researchers can better link general physical descriptors (i.e., emaciated, skinny, normal, fat) that are normally related to body condition values [21] with actual individual health status, to better inform management and decision-making in the context of restoration. To this end, we assessed the relationship between alligator body condition and 36 hematological and biochemistry (blood) parameters (S1 Table) related with general health, nutrition, dehydration, stress, and inflammation/infection in four areas (Arthur R. Marshall Loxahatchee National Wildlife Refuge—LOX, Water Conservation Area 3 -WCA3, Everglades National Park -ENP, and Florida Panther National Wildlife Refuge -Panther) across two physiographic regions (Everglades and Big Cypress) of the Greater Everglades, South Florida (United States). We aimed to answer two main questions, 1) are there any alligator blood parameters that strongly relate to body condition that can validate its use as a descriptor of nutritional physiology and overall health? 2) is there a spatial difference between physiographic regions and areas in the quantity and type of blood parameters that relate with body condition in the Greater Everglades? We hypothesize that blood parameters, mainly those related with nutrition, will relate to body condition as they reflect food intake quantity and quality [8–10]. We also expect that blood parameters highly associated with health conditions (general health and inflammation/infection) will be related to body condition as general health may influence an alligator’s ability to forage or reflect generally unfavorable conditions [8–10, 13]. Finally, we expect that relationships between blood parameters and body condition will follow a spatial pattern reflective of the different physiographic regions because of differences in hydrology and prey production and availability [12].

## Materials and methods

### Study area

**Fig 1:**
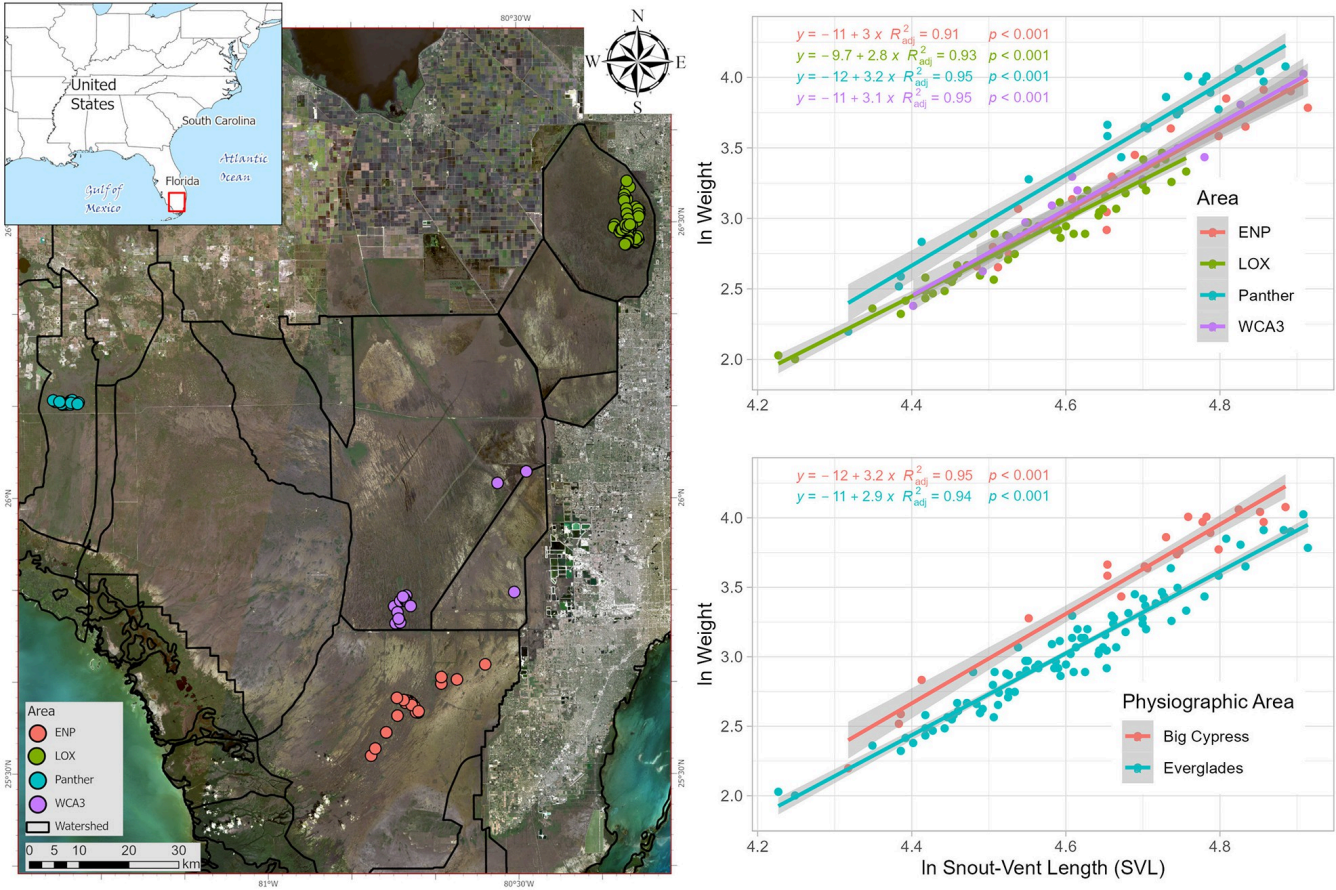
Study area and alligator natural log-transformed (ln) weight and ln snout vent length (SVL) regression models. Notice the differences in slope between the Big Cypress region represented in this analysis by the Florida Panther National Wildlife Refuge (Panther) and the rest of the areas included in the Everglades region (LOX = Arthur R. Marshall Loxahatchee National Wildlife Refuge, WCA3 = Water Conservation Area 3, and ENP = Everglades National Park). These differences in slope mean that there is a weak effect (p-value < 0.1) of where animals were captured on the weight ~ SVL relationship. In simple words, this means that alligators in Panther are heavier than alligators found across the Everglades region relative to body length. Statistical results that corroborate these differences can be found in Table 1. All alligators captured in the study were ≥ 125 cm TL and were captured from 2017 to 2019. The points on the map refer to each alligator captured per area.

This study covered four main areas across two physiographic regions (Everglades = LOX, WCA3, and ENP, and Big Cypress = Panther) that are representative of the current Greater Everglades (Fig 1). In the most northeastern part of the Everglades region is LOX (Palm Beach County) comprising 573 km^2^ of wetlands surrounded by canals and levees with a north to south water depth and hydroperiod (number of days out of the year water depth is > 0) gradient that did not occur before landscape modifications in the 1950s [22]. Marsh water depths are shallower in the north, deeper in the south, and follow a seasonal pattern with the highest water depths in October (end of wet season) and lowest water depths in May (end of dry season) [22]. In the center of the Everglades region is WCA3 (Broward and Miami-Dade counties) comprising 2,369 km^2^ of marshes surrounded by canals, levees, and water control structures. It is managed for multiple uses including flood protection, water supply, recreation, and fish and wildlife [23]. Currently, this area is primarily rainfall driven, receiving up to 60% of its hydrologic input from rain with, similar to LOX, a north to south water depth—hydroperiod gradient that did not occur before landscape modifications (northern portion drier than historic while southern portion is wetter than historic because water backs up behind levees) [23]. In the southernmost portion of the Everglades is ENP, comprising ~ 5,700 km^2^ and characterized by a very low relief naturally composed of marshes (peat and marl wet prairies and sawgrass), sloughs, ridges, and tree islands as well as few man-made ponds, canals, and ditches [24–26]. Landscape modifications (roads, levees, and canal construction) and water management occurring north of this area has affected the natural water flow of the system, exposing some parts of ENP to unnatural and prolonged dry conditions and has caused negative effects on biodiversity [25]. Finally, Panther Refuge (Collier County) is 106 km^2^ and is located on the central west area of the Greater Everglades in the Big Cypress Basin, which includes the northern origin of the Fakahatchee Strand, the largest cypress strand in the Big Cypress swamp [27]. Vegetation in Panther is a mosaic of wetlands (cypress swamps and wet prairies) and uplands (hardwood hammocks and pinelands) providing a variety of habitats for a rich species diversity. Water depths vary seasonally with shallow waters generally present in mid-May and deep waters present in mid-October [27].

### Fieldwork and sampling

We captured and collected samples from wild alligators greater than or equal to 125 cm total length (TL) inhabiting marsh habitats in LOX, WCA3, and ENP, and lake/swamp habitats in Panther at night in October and November 2017, April, September, October, and November 2018, and March and April 2019 using standard protocols [28]. We chose this size cutoff to avoid confounding effects from smaller size classes (hatchlings and juveniles) on body condition. Captures were done using airboats across all areas except Panther where we captured alligators by swamp buggy, canoes, or foot from the edge of the tram trail. All animals were captured using snares and brought onboard or to land as soon as possible (see results) to collect blood samples and measurements to minimize stress effects on blood values [17]. We did not capture animals in summer (May through August) to circumvent unwanted effects due to reproductive behavior (mating and laying eggs) on blood parameters. Samples were withdrawn from the ventral coccygeal vein below the transverse process of tail vertebrate using a 20 G x 1.5-inch Luer-Lok tip with a 6 mL syringe (BD Medical Company, New Jersey, USA). Samples were transferred either into a 3 mL BD Lithium Heparin Vacutainer (BD Medical Company, Franklin Lakes, NJ) or a microvette 500 μL Lithium Heparin vials (Starsted Inc., Nümbrecht, Germany) and inverted several times to ensure even mixing. Vials were stored in a cooler with an insulating layer to avoid direct contact of samples with ice, minimizing the risk of blood cells lysing [29]. Total length (TL), snout-vent-length (SVL), weight, sex (cloacal digital palpation), and capture location were also recorded. Animals were released at their capture site after sample and data collection. This study was done under University of Florida Institutional Animal Care and Use Committee (IACUC) protocol # 201509072, Arthur R. Marshall Loxahatchee National Wildlife Refuge Research and Monitoring Special Use Permit 6B16-003, Florida Panther National Wildlife Refuge Research and Monitoring Special Use Permit 41545-201705R, Florida Fish and Wildlife Conservation Commission Scientific Collecting Permits SPGS-13-58 and SPGS-17-62, and Department of the Interior National Park Service Scientific Research and Collection Permit EVER-2016-SCI-0014.

### Hematology and biochemistry analysis

Blood samples were transferred within 12 hours of collection to the University of Miami’s Comparative Pathology laboratory, Florida, USA for analysis. Blood smears were prepared on full slides and complete blood counts (CBC; 100 cells) with differential were performed using the Natt and Herrick method [30] with Wright Giemsa stain at 1,000X (microscopic magnification). Natt and Herrick’s stain (Vetlab, Miami, Florida, USA) and a hemacytometer with improved Neubauer counting chamber (VWR International, Radnor, Pennsylvania, USA) were used for absolute (abs) white and red blood cell counts. Blood plasma was obtained by spinning samples for 5 minutes at 10,000 rpm (9391g) in an Eppendorf 5254 centrifuge (Eppendorf AG, Hamburg, Germany). Plasma biochemistry was performed using Vitros 250 (Ortho, Rochester, New York, USA), bile acids and hydroxybutyrate reagents (Randox, Kearneysville, West Virginia, USA) with RX Daytona (Randox). Total protein, albumin, alpha-1 globulins, alpha-2 globulins, beta globulins, and gamma globulins were measured via electrophoresis using Helena reagents and SPIFE 300 analyzer (Helena, Beaumont, Texas, USA). All analyses were done within 24 hours of sample collection except for corticosterone, which was completed in batches at the end of the sampling season from plasma banked at -80°C. Samples were rejected if clotted, diluted, suspected to contain lymph, or with a high hemolysis (≥ 2) index values because the effect these variables had on chemical plasma composition [31]. Finally, biochemistry values reported as outside the instruments range (e.g., triglycerides values < 9 and > 575) were excluded from analysis due to the uncertainty of the actual value.

### Body condition calculation

For all following analyses, we defined statistical evidence based on critical values (p-value) as very strong (≤ 0.001), strong (≤ 0.01), moderate (≤ 0.05), weak (≤ 0.10) or little-to-no evidence (> 0.10) as suggested by Muff [32]. Uncertainty within models is depicted as standard errors. All statistical analyses were performed in R [33].

We assessed the linear relationship between natural log-transformed (ln) weight and ln SVL via ordinary least square (OLS) multiple linear regression models (*lm* function) using physiographic region, area, and sex as covariables to evaluate any confounding effects (change in slope) when calculating body condition that could bias the signal derived from blood parameters. This approach allowed us to separate intrinsic variation of the data (natural dispersion of the data found in the population analyzed) and observed variation caused by covariables. We did this by testing the levels of variability within regression models by covariate via *anova* and comparing estimated marginal mean slopes via *lstrends* (“emmeans” package) functions [34]. Data that showed no evidence of an effect of covariables on slopes were analyzed together (see results). We calculated relative body condition of alligators based on the relative condition factor (relative K = Mass / SVL^slope^) [35] using untransformed weight and SVL data and the estimated slope derived from physiographic regions (slopes = Everglades 2.95 ± 0.08 and Big Cypress 3.22 ± 0.16; see results) multiplied by 10^4^ (scale factor). This implies that relative K individual numbers cannot be compared directly between physiographic regions due to the difference in slopes (allometry coefficients). However, differences in slope can inform that, given a size, a group of animals with a larger slope weight more than other with a lower slope (Fig 1). These results can also be analyzed to look for trends and relationships as an indication of effect from blood parameters, which was the main objective of the present study.

### Body condition—Blood parameters relationship

For each physiographic region we ran single OLS linear regression models with relative K as response variable and blood parameters as independent (predictor) variables individually to determine linearity (very strong to weak evidence), heteroscedasticity (spearman rank correlation analysis -*cor*.*test* function and residual-fitted plots), and presence of outliers (studentized deleted residuals -*studres* function, “MASS” package; S1 Table) [36]. Observations for which the studentized deleted residuals exceeds 3 were not included in the analysis, so that the final statistical model describes the dominant pattern in the data [37]. We ran multiple OLS linear regression models including all blood parameters as well as grouping them by categories (general health– 4 variables, nutrition– 13 variables, dehydration– 8 variables, stress– 1 variable, and inflammation/infection– 12 variables). When predictors did not show evidence of an effect on the response variable, a new multiple regression model was run only including significant predictors. We tested whether models with significant predictors fit equal or better than models with all predictors by groups via the *linearHypothesis* function from “car” package [38]. We selected the best model using Akaike Information Criteria (AIC) via *aictab* function from the “AICcmodavg” package [39]. Finally, we assessed model performance (model accuracy) by estimating the expected prediction error via repeated k-fold cross-validation analysis (10-folds and 3 repeats) using the *train* function from the “caret” package [40]. We ran this analysis on models where evidence of an effect was found (individual predictors) as well as the best models selected by AIC. We used the squared correlation between observed and predicted values by the model (R^2^) as a measure of variance (how much variation is capture by the model) and the mean absolute error (MAE, the average absolute differences between observed and predicted outcomes) as a measure of accuracy. This process was repeated by area to evaluate whether intrinsic habitat conditions could influence blood parameters as descriptors of alligator body condition. Repeated k-fold cross-variation predictability range was estimated based on the standard deviation of R^2^ estimated per fold and repeat.

## Results

We collected blood samples and morphometric data from 120 American alligators in LOX (59), ENP (21), WCA3 (15), and Panther (25). Of those, 25 samples were drawn from adults (≥ 175 cm), 95 from subadults (≥ 125 cm to < 175 cm), 79 from females, and 41 from males; 72 alligators were captured in fall (September—November) and 48 in spring (March—April). Only one individual was captured twice in Panther (April and September 2018) during the study. Alligators ranged from 136.2 cm to 270.6 cm total length (mean = 200.9 cm), 68.5 to 136.2 cm SVL (mean = 102 cm), and 7.40 to 59 kg weight (mean = 25.93 Kg). We excluded two samples from analyses due to high levels of hemolysis index values (≥ 2) and only used the most recent data from the alligator that was recaptured in Panther for analysis (final n = 117). Time between capture and blood draw was on average 11.02 ± 5.70 minutes ranging from 10.03 ± 4.95 min in LOX to 13.00 ± 7.29 in Panther.

### Body condition calculation

We found no evidence of an effect neither by sex nor by area on ln weight ~ ln SVL slopes (allometric coefficient) except when including all areas together (moderate evidence of an effect by sex) and when comparing specifically body condition in LOX and Panther (weak evidence of an effect by area; Table 1). We also found weak evidence of an effect of physiographic region on the allometric coefficient. It is important to notice that adults and subadults were included together for analysis as the number of adults per area did not allow us to test for an effect of this variable (size group) on the body condition per area. However, all models groping both size groups showed low variation and high descriptive power (R^2^ > 90%).

**Table 1:** Analysis of variance (ANOVA) and contrasting marginal mean slope analysis for American alligators by physiographic region, area, and sex.

Contrast	Estimate	Standard Error	Degrees of Freedom	t-ratio	p-value
ENP F—M	0.27	0.74	17	0.36	0.72
LOX F—M	0.10	0.23	55	0.44	0.66
Panther F—M	0.08	0.41	18	0.19	0.85
WCA3 F—M	-0.30	0.50	11	-0.60	0.56
Everglades F–M	-0.13	0.18	91	-0.74	0.45
Big Cypress F—M	0.08	0.41	18	0.19	0.85
All together F—M	0.46	0.22	113	2.10	**0.04**
ENP—LOX	0.23	0.21	109	1.11	0.69
ENP—Panther	-0.23	0.22	109	-1.06	0.71
ENP—WCA3	-0.07	0.27	109	-0.27	0.99
LOX—Panther	-0.46	0.18	109	-2.57	**0.06**
LOX—WCA3	-0.30	0.24	109	-1.27	0.58
Panther—WCA3	0.16	0.25	109	0.65	0.91
Big Cypress—Everglades	0.27	0.16	113	1.68	**0.09**

Bold numbers refer to models in which we obtain at least weak evidence of an effect of covariates on ln weight ~ ln SVL relationship. All alligators captured in the study were ≥ 125 cm TL and were captured from 2017 to 2019. LOX = Arthur R. Marshall Loxahatchee National Wildlife Refuge, ENP = Everglades National Park, WCA3 = Water Conservation Area 3, Panther = Florida Panther National Wildlife Refuge, F = female, M = male. Notice the lack of evidence of an effect of covariates except when combining all areas together by sex and when pairwise LOX and Panther.

Based on these results, we calculated body condition by physiographic region as it was the minimum unit in which we did not have confounding effects using a slope of 2.95 for the Everglades region and 3.22 for the Big Cypress region (Fig 1). This difference in allometric coefficient means that Panther alligators weighed more than any other alligator captured in any of the areas within the Everglades (ENP, LOX, and WCA3) when standardized by SVL (Fig 1), which reflects the larger slope obtained for this area/region. Within the Everglades region, relative K calculated based on the same allometric coefficient showed that alligators captured in WCA3 (relative K 2.75 ± 0.27) and ENP (2.72 ± 0.12) weighed on average more / had higher body condition values than alligators captured in LOX (2.59 ± 0.24; Fig 1).

### Body condition—Blood parameters relationship

Most of the regression models showed no evidence of heteroscedasticity (ρ close to 0, p value > 0.10) except for some instances where weak evidence was present (S1 Table). The most common number of outliers found across models was one, which was removed before modelling. All prealbumin values from alligators captured in ENP and WCA3 and blood urea nitrogen values from alligators captured in WCA3 were constant across all animals (zero and one, respectively) so these blood parameters were not included in models. Model results are presented by physiographic region because they have different allometric coefficients (slopes).

#### Everglades region

**Fig 2:**
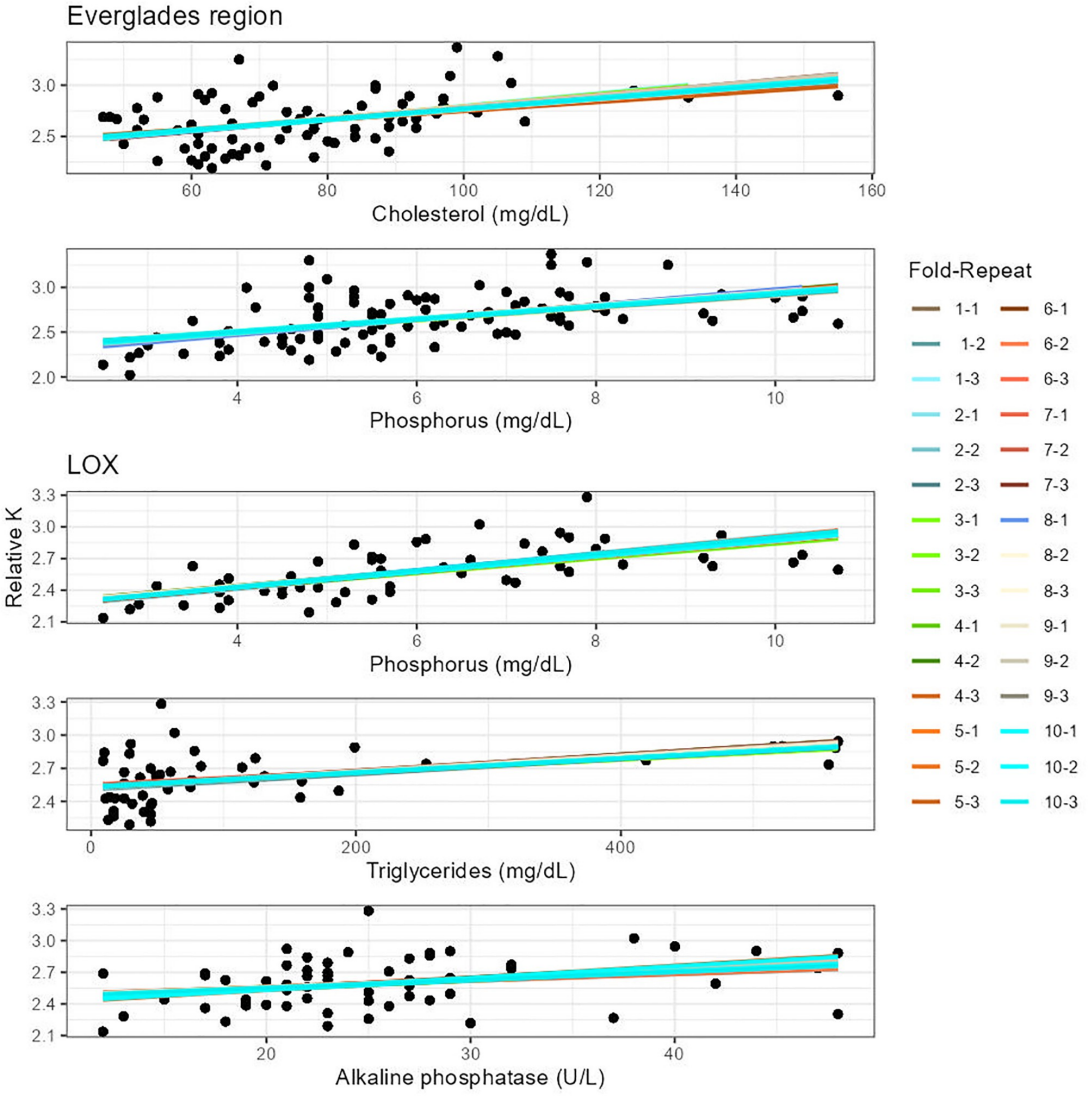
Cross validated models for single American alligator blood parameters. We present here a comparison between single blood parameters that perform best for the whole Everglades region and Arthur R. Marshall Loxahatchee National Wildlife Refuge (LOX). Alligators included in this analysis were ≥ 125 cm TL and captured from 2017 to 2019. Each linear regression model per graph refers to each fold and repeats within the repeated k-fold cross validation analysis. We present only predictors with the higher predictability levels (see Table 2 for details).

We found very strong to weak evidence of a linear relationship between 19 blood parameters and relative K in the Everglades with models accounting for between 2 and 27% of the variance (S1 Table). However, repeated k-fold cross-validation analysis showed that most of these models are highly variable in their predictability (i.e., anion gap ranging from 0 to 38% of variance explanation), reducing the number of predictive models that account for more than 12% and up to 52% body condition variance to two (phosphorus and cholesterol; Table 2, Fig 2). Both blood parameters have a MAE of 0.18 relative K units, which is about one eighth of the relative K range for the Everglades region (2.02–3.37).

**Table 2:** Repeated k-fold cross-validation analysis including American alligator hematological and biochemistry parameters affecting body condition.

	Everglades												Big Cypress		
	LOX			WCA3			ENP			All			Panther		
Blood parameter	R^2^	Range	MAE	R^2^	Range	MAE	R^2^	Range	MAE	R^2^	Range	MAE	R^2^	Range	MAE
Heterophils abs	0.27	0.05–0.49	0.18												
Eosinophils abs							0.93	0.71–1.15	0.26	0.17	-0.01–0.35	0.22			
Alkaline Phosphatase U/L	0.38	0.13–0.63	0.18							0.16	0.01–0.32	0.22			
Amylase U/L	0.29	0.08–0.49	0.18							0.20	0.06–0.35	0.21	0.95	0.8–1.1	0.10
Alanine aminotransferase U/L	0.26	-0.03–0.55	0.19							0.19	0.01–0.37	0.21			
Aspartate Aminotransferase U/L							0.98	0.86–1.09	0.28				0.92	0.73–1.11	0.10
Calcium mg/dL	0.31	0.04–0.59	0.17							0.11	0.02–0.2	0.21			
Cholesterol mg/dL	0.42	0.12–0.73	0.17	1.00	0.00	0.23				0.29	0.12–0.45	0.18	1.00	1–1	0.08
Lipase U/L	0.31	0.05–0.58	0.19							0.12	-0.06–0.3	0.22			
Magnesium mg/dL				1.00	0.00	0.16									
Sodium mmol/L	0.24	0.01–0.47	0.19							0.15	-0.03–0.32	0.22	0.89	0.62–1.16	0.09
Chloride mmol/L	0.24	0.01–0.47	0.19												
CO2 mmol/L										0.16	-0.03–0.34	0.21			
Glucose mg/dL	0.26	0–0.51	0.18							0.14	-0.01–0.29	0.22			
Anion Gap mmol/L	0.36	0.08–0.64	0.17							0.19	0–0.38	0.20			
Osmolality (Calc)													0.93	0.7–1.16	0.10
Phosphorus mg/dL	0.57	0.34–0.79	0.14				0.94	0.74–1.15	0.29	0.32	0.12–0.52	0.18			
Calcium–Phosphorus ratio	0.42	0.09–0.74	0.16				0.94	0.73–1.16	0.28	0.28	0.09–0.47	0.20			
Uric Acid mg/dL							0.96	0.77–1.14	0.26				0.95	0.76–1.15	0.08
Total Proteins, g/dL	0.34	0.05–0.63	0.18							0.19	0–0.37	0.21			
Triglycerides mg/dL	0.44	0.14–0.74	0.16	1.00	0.00	0.23	-	-	0.17	0.25	0.02–0.48	0.19			
Albumin abs	0.27	0.03–0.5	0.18							0.16	0–0.32	0.21			
Alpha 1 Globulins abs	0.30	0.05–0.56	0.17	1.00	0.00	0.16				0.20	0–0.4	0.20	0.90	0.65–1.14	0.11
Alpha 2 Globulins abs	0.26	-0.02–0.54	0.18												
Beta Globulins abs	0.28	0.04–0.52	0.18												
Gamma Globulins abs	0.29	0.06–0.51	0.18							0.19	-0.02–0.39	0.21			
Hydroxybutyrate, mmol/L	0.25	-0.03–0.54	0.19							0.13	-0.03–0.3	0.22			

This table presents the prediction error (MAE = mean absolute error), the variance explained by the model (R^2^), the standard deviation of the variance (R^2^ SD), and the range of the variance after running 10-folds three times. All alligators included in this analysis were ≥ 125 cm TL and were captured from 2017 to 2019. Abs = absolute counts. LOX = Arthur R. Marshall Loxahatchee National Wildlife Refuge, ENP = Everglades National Park, WCA3 = Water Conservation Area 3, Panther = Florida Panther National Wildlife Refuge.

**Fig 3:**
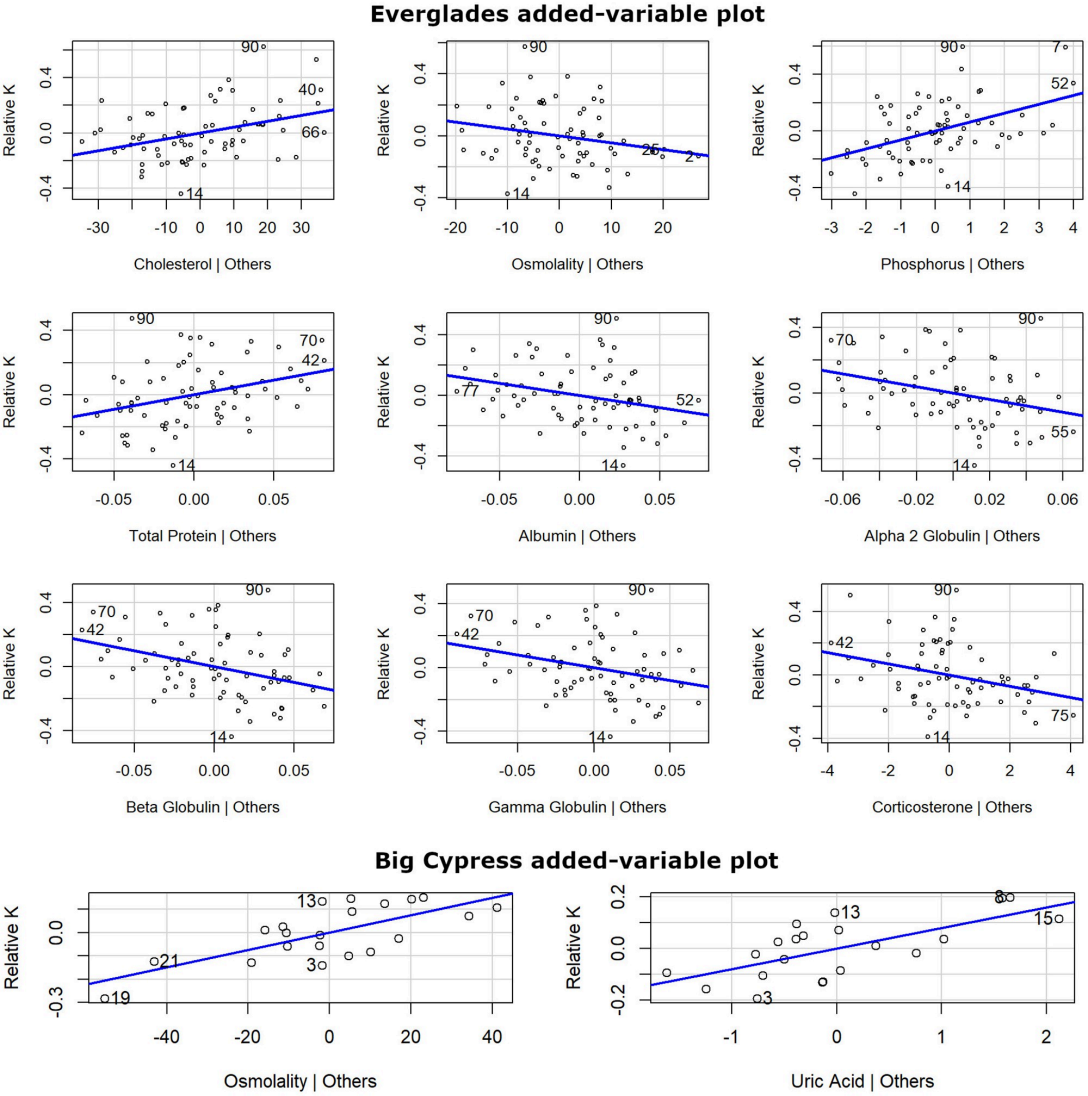
American alligator partial regression plots for Everglades and Big Cypress regions. Models used body condition factor (relative K) as the response variable and the best performing hematological and biochemistry parameters based on the Akaike Information Criteria (AIC) selection as predictor variables (statistical information in Table 2). Values on both axes are residuals derived from regressing the response variable against independent variables without the target variable (Y; e.g., cholesterol) and regressing the variable of interest against the remaining independent variables (X). All alligators included in this analysis were ≥ 125 cm TL and were captured from 2017 to 2019.

The best combination of blood parameters (predictors) that can explain up to 40% of the variation found in alligator body condition for this region were cholesterol (positive related, nutrition group), phosphorus (positive), osmolality (negative), total protein (positive), albumin (negative, dehydration group), alpha 2, beta, and gamma globulins (negative; inflammation/infection group), and corticosterone (negative, stress group; AIC = -14.52; Table 3, Fig 3). When cross-validated, this model has a MAE of 0.16 relative K units and accounts for 37 ± 21% of the variance. The next two models which had an ΔAIC of less than 2, included all the aforementioned variables plus magnesium explaining 41% of the variation (cross-validation = 40 ± 21%, MAE = 0.16) and the model that isolated blood parameters related with nutrition explaining 30% of the variation (cross-validation = 34 ± 19%, MAE = 0.18). These top three models provide a cumulative model weight (predictive power) of 89%. None of the variables categorized as general health (red blood count, CO_2_, blood urea nitrogen -BUN, and uric acid) showed evidence of linearity when analyzed as a group. However, some of them showed evidence of linearity when analyzed individually (e.g., CO_2_; S1 Table).

**Table 3:** Multiple regression analysis models classified based on importance via Akaike Information Criteria (AIC).

Model names	K	AICc	ΔAICc	AICc Weight	Log-Likelihood	Cumulative Weight
*Everglades*						
All significant parameters	11	-14.52	0.00	0.42	20.50	0.42
All significant + magnesium	12	-13.86	0.66	0.30	21.62	0.72
Nutrition all significant	5	-12.73	1.79	0.17	11.78	0.89
Nutrition/Dehydration	5	-10.63	3.89	0.06	10.74	0.95
All significant + ALT, BUN, and Tri	15	-10.13	4.39	0.05	24.43	1.00
*Big Cypress*						
All significant parameters	4	-35.30	0.00	0.80	22.90	0.80
All significant + phosphorus	5	-32.42	2.88	0.19	23.21	0.99
Nutrition	5	-25.76	9.53	0.01	20.19	1.00
*LOX*						
Dehydration all significant	5	-28.94	0.00	0.68	20.03	0.68
Nutrition/Dehydration all significant	5	-26.36	2.57	0.19	18.91	0.87
Dehydration filtered	7	-25.54	3.40	0.12	20.87	0.99
Nutrition/Dehydration	7	-17.34	11.59	0.00	17.32	1.00
*ENP*						
Nutrition selected parameters	5	12.12	0.00	0.92	1.44	0.92
Inflammation/Infection filtered	5	17.03	4.91	0.08	-1.51	1.00
*WCA3*						
Dehydration filtered	6	3.79	0.00	0.99	9.35	0.99
Nutrition selected parameters	5	13.94	10.15	0.01	2.31	1.00

Values are reported per region (Everglades and Big Cypress) and area (LOX = Arthur R. Marshall Loxahatchee National Wildlife Refuge, ENP = Everglades National Park, and WCA3 = Water Conservation Area 3). Models used American alligator body condition factor (relative K) from animals ≥ 125 cm TL captured 2017–2019 as response variable and hematological and biochemistry parameters as predictor variables. This table contains the top models until reaching 100% cumulative weight because the rest of the models (20+) did not provide any new information (prediction power) to the analysis. Detailed information about variables included in models can be found in S2 Table.

When data were analyzed by area within the Everglades region, we found a different set of variables that related linearly with body condition and that explained the most variation of the response variable. For instance, in LOX we found very strong to moderate evidence of linearity between 21 blood parameters and relative K, whereas for ENP and WCA3, only five and four parameters respectively showed strong to weak evidence of linearity with relative K (S1 Table). The most relevant individual blood parameters for LOX were anion gap and phosphorus, explaining 29 and 46% of the body condition variation respectively, while for ENP uric acid and triglycerides explained 26 and 46% of the variation and for WCA3 magnesium and alpha 1 globulin explained 22 and 32% of the variation. However, when cross-validated, we found that models from WCA3 and ENP overpredict body condition values due likely to model instability because low sample size (15 ± 1 and 20 ± 2, respectively). In the case of LOX, cross-validated models showed phosphorus, triglycerides, and alkaline phosphatase as the best performing models, explaining more than 13% and up to 79% of the body condition variation (Table 2). Phosphorus had the lowest prediction error (0.14 relative K units), followed by triglycerides (0.16), and alkaline phosphatase (0.18).

**Fig 4:**
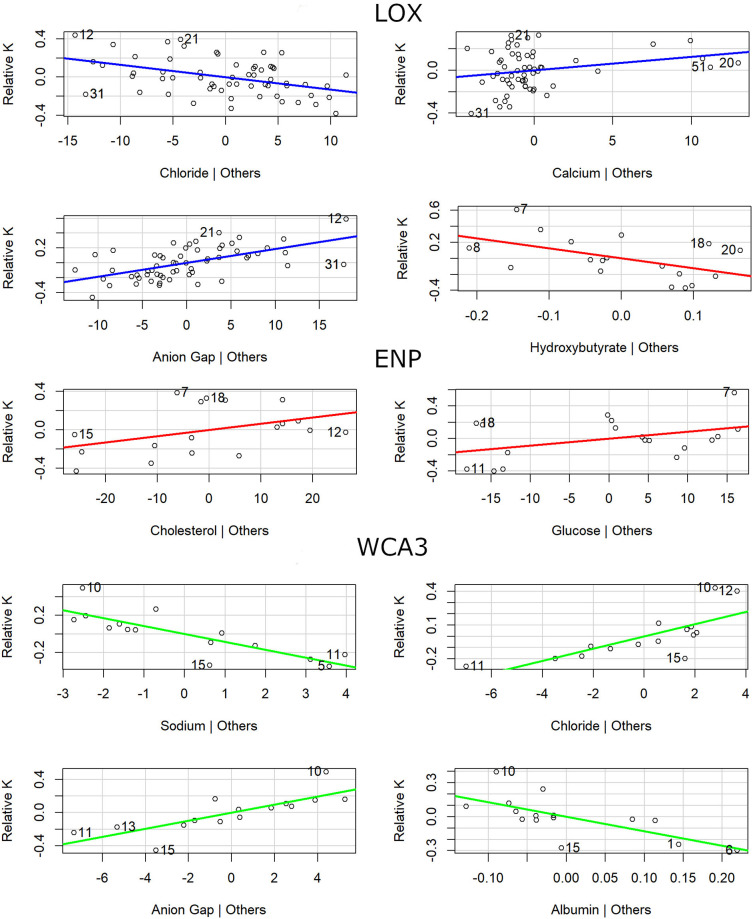
American alligator partial regression plots by capture area. Models were run for Arthur R. Marshall Loxahatchee National Wildlife Refuge (LOX, blue), Water Conservation Area 3 (WCA, green) areas, and Everglades National Park (ENP, red). Models include alligator body condition factor (relative K) from animals ≥ 125 cm TL captured 2017–2019 as response variable and the best performing hematological and biochemistry parameters based on the Akaike Information Criteria selection (statistical information in Table 2).

We found that the best combination of blood parameters that can explain up to 44% of the variation (52 ± 24% when cross-validated) found in alligator body condition at LOX were a smaller pool of variables (chloride negative related, anion gap positive related, and calcium positive related; AIC = -28.94; Fig 4) with a smaller predicted error (MAE = 0.15) compared with the whole Everglades region. This model by itself carried 68% of the total cumulative model weight, compared with the 42% found in the best model for the whole Everglades. Interestingly, the second best model in LOX (AIC = -26.36) that combines blood parameters related with nutrition and dehydration such as cholesterol (positive related), chloride (negative related), and anion gap (positive related) can explain up to 50% (58 ± 29% when cross validated) of the variation in body condition and increase the cumulative model weight up to 87%, which is more than what can be explained by the best model combining all Everglades data together (Table 2). The same pattern was found in ENP where the best model included only cholesterol (positive related), glucose (positive related), and hydroxybutyrate (negative related; AIC = 12.12) but only explained 15% of the body condition variation (94 ± 20% when cross-validated) with a predicted error of 0.23 relative K unites and carried 92% of the cumulative model weight. The largest percentage of explanation from a group was found in WCA3 (66%) and included dehydration blood parameters (chloride and anion gap positive related and sodium and albumin negative related) and carried 99% of the cumulative model weight. However, models from ENP and WCA3 could not be cross-validated due to sample size effect in model stability.

#### Big Cypress region

We found strong to weak evidence of linearity between 7 blood parameters and relative K for the Big Cypress region but, in comparison with our results from the Everglades region, all 7 parameters individually explain between 10 (e.g., amylase) and 41% (uric acid) of the variation of the data (S1 Table). However, repeated k-fold cross-validation analysis showed that all 7 models overpredict body condition values (i.e., R^2^ above 100%) likely due to low sampling (mean sample size 21.3 ± 1.1), which could cause model instability (Table 2). Mean absolute error (MAE) for these models varied between 0.08 and 0.11 relative K units, which is about one quarter of the relative K range for the Big Cypress region (0.83–1.22). Multiple regression models were limited to a maximum of 14 predictors for this region due to the reduced sample size. Nonetheless, the best combination of blood parameters included osmolality (dehydration) and uric acid (general health) both positively related, explaining 49% of the variation in body condition and carrying 80% of the cumulative model weight (AIC = -35.30; Table 3; Fig 3). When cross-validated, this model has a MAE of 0.09 relative K units and accounts for 89 ± 26% of the variance.

## Discussion

Our study provides, for the first time, cross-validated evidence of the predictive power of alligator blood parameters on body condition, determining what seems to be the most relevant blood parameters for the Greater Everglades at the time sampling was performed (phosphorus, cholesterol, triglycerides, and alkaline phosphatase) from a list of 36 hematological and biochemistry parameters, as well as pointing out the relevance of the scale at which analysis are done (local or regional) for power prediction. While most of the models had a wide predictability range depending on the blood parameter used (~ ± 26%) and the scale at which analysis had been conducted, prediction error for the whole Everglades region as well as for LOX were as low as one eighth of the relative K range defined for those areas, which provides an initial uncertainty measure for these predictive models. Also, although we were not able to cross validate models from Panther, WCA3, and ENP because of effect of sample size on model stability, these areas showed a completely different set of blood parameters linearly related to body condition compared to LOX and general Everglades regions, which could imply a stronger habitat effect or even a spatial dependent relationship. These results partially support our hypothesis not only showing the relevance (as expected) of nutrition parameters and in some specific cases, depending on the area, inflammation/infection parameters, but also providing new information on parameters that are also relevant for body condition (e.g., phosphorus). Further research with a much larger sample size in these areas (Panther, WCA3, and ENP) could test more in depth these hypotheses and shed light on the spatial variability of body condition ~ blood parameters relationship in the Greater Everglades. Also, testing these hypotheses in captive populations (which tend to have greater body conditions) can allow us to identify whether the same sort of blood parameters or a new set of them are linearly related with body condition under those conditions [41].

The Greater Everglades has faced dramatic landscape changes in the last two centuries due to hydrological management that has allowed for expansion of human population settlements and agricultural development, disrupting ecological interactions (e.g., food webs) as well as structurally and functionally affecting wildlife populations [25]. Effects of these changes on wildlife have been well documented in alligators, where population attributes such as birth rate (clutch size), abundance, and population body condition have shown negative trends since first documented [11, 12, 42–44]. In 2000 the Comprehensive Everglades Restoration Plan was authorized by Congress to implement restoration efforts to bring back more natural hydrological conditions to the ecosystem. Restoration of more natural hydrologic conditions is expected to result in improvement of population parameters for alligators and other species [45]. In this context, our results indicated that several hematological and biochemistry parameters are linearly related to body condition and that the level of explanation increased as we combined them in a meaningful/diagnostic way (e.g., blood parameters related with nutrition and dehydration are the best body condition predictors for Everglades), providing valuable information for management. However, results also indicated that different areas could have different blood parameters that better describe the variation found in alligator body condition in the Everglades, which implies a tighter relationship between habitat conditions and body condition than previously thought. This variation in signals across areas highlights the relevance of focusing on a complete medical panel rather than a couple blood parameters when assessing broad areas such as the Greater Everglades.

Although several hematological and biochemistry reference intervals for American alligators are currently available [14], these ranges were estimated based on the same data presented in this study, making them unusable (due to circularity) in our context. Despite this, it is reasonable to imply that alligators with better body condition in the Greater Everglades are within the biological optimal range for a given parameter. This means that alligators with lower body condition are likely experiencing lower than ideal values for a given parameter when the relationship is positive, and higher than ideal values for that parameter when the relationship is negative. Based on this rationale, a fair interpretation of the variation found across areas based on multiple regression analysis could be that when LOX and ENP alligators are in poor body condition it is likely due to dehydration and inadequate diet (likely hypocalcemia and hypolipidemia) whereas when WCA3 alligators are in poor body condition it is likely due to dehydration (likely hypernatremia and hyperalbuminemia). These trends become pooled when looking at the nine parameters deemed significant for the total Everglades group (both dehydration and inadequate diet), but with the addition of the Alpha2/Beta/Gamma globulins indicating there is additional antigenic stimulation/infection risk impacting this population. Interestingly, in the event of dehydration and malnutrition occurring simultaneously, animals with dehydration may be masking an even more profound protein deficiency. All this information derived from our methodological approach is highly relevant in a management framework because we are not only finding a clear relationship between these two sets of variables (body condition and blood parameters), but also gaining information on how individual populations are coping with the environment and how different stressors play different roles spatially even in the same ecosystem.

One interesting result was the strong relationship found between phosphorus and body condition in the Everglades, especially in LOX, likely influenced by prey intake (low body condition–low prey intake—low phosphorus values; high body condition–high prey intake–normal phosphorus values), which may show an active bioaccumulation process across the food web. Nonetheless, the lack of reference intervals to define whether alligator phosphorus values (or values on any prey) in the Greater Everglades are higher than normal limits conclusions. One of the few studies on alligator blood parameters in South Carolina reported a mean phosphorus of 5.3 mg/dL in juvenile alligators ranging from 4.2 to 7.5 mg/dL [7], which is below the mean phosphorus reported in our study (6.43 mg/dL) with also a smaller range compared with our results (2.0–15.6 mg/dL). Phosphorus is a mineral macroelement involved in a wide range of physiological and biological processes from skeletal formation to animal metabolism [46]. In a mature animal, it is estimated that 80% of its total body phosphorus is contained in bones [46]. In the Greater Everglades, phosphorus is naturally limited, making the system extremely oligotrophic and sensitive to small increases in phosphorus concentration [47]. However, agricultural practices have impacted phosphorus concentration increasing it to unnatural levels across the system. Work done in the last 30 years in to reduce phosphorus concentration from the agricultural area into the Greater Everglades has helped to reduce phosphorus values in the water closer to what appears to be normal levels (< 100 mg/L) [48]. Whether alligators are potentially acting as phosphorus sink traps, retrieving, and keeping high levels of phosphorus out of the system is a relevant question that should be tested to assess potential health effects in alligators as excess of blood phosphorus affect calcium absorption resulting in retarded growth, high basal metabolic rate, eggshell thinning, and reduce activity and sensitivity [46].

Blood parameters such as cholesterol and triglycerides have been previously used as indicators in evaluation of nutrition as well as been associated with feeding status in reptiles [31, 49, 50]. Previous research has also indicated that glucose concentrations are contingent on nutritional status and diet in reptiles [7, 51]. These studies along with our results suggest that body condition could be indicative of feeding and nutritional status in alligators and can be related with health status when prey availability is a limiting factor. This concept is further reinforced by research demonstrating that cholesterol and triglyceride levels increase during active periods compared to hibernating periods in Chinese alligators (*Alligator sinensis*) [52]. In our study, cholesterol, triglycerides, and glucose were different when compared between alligators captured in Panther (cholesterol 148 ± 44 mg/dL, triglycerides 257 ± 207 mg/dL, and glucose 104 ± 47.7 mg/dL) and LOX (77.4 ± 23.7 mg/dL, 122 ± 163 mg/dL, and 66.3 ± 12.7 mg/dL), likely implying these two areas highly differ in prey availability/quality. Panther refuge is part of the Big Cypress ecosystem and alligators in this area may have more access to mammalian prey items such as deer, hogs, and marsh rabbits which are known to be a better source of nutrition [53, 54]. In contrast, areas such as LOX may have more abundant aquatic than terrestrial prey due to topography and water depths, which influences food quantity and quality. Alligators require prey not only to be produced (which normally happens in wet season) but also concentrated as water level drops in the beginning of the dry season [12]. If these conditions are altered due to either water management or natural conditions (abnormal dry seasons) prey quality, quantity, and availability will be highly affected which will be reflected in alligators’ body condition [44].

Overall, our results showed that there are not only several biochemistry and hematological parameters that were related with alligator body condition, providing a way to validate the latter as a health descriptor (mainly describing nutritional physiology and physical fitness), but that these relationships are highly influenced by where alligators live. We also found that body condition could be influenced by area as well as population parameters such as sex, which must be factored in when running body condition analysis to better understand the intrinsic variation of the data. Interestingly, not all blood parameters relate to body condition in the same way. We recommend a more in-depth analysis be conducted to understand what specific characteristics are influencing body condition in a defined area. This means that although alligators from similar areas could have similar body conditions under the same allometric coefficient, it is possible that the factors that are influencing body condition in each area could be different (i.e., low food intake vs sickness). Thus, hematological and biochemistry values provide a useful tool for evaluating health and nutrition in American alligators as well as provide insights about the current environmental stressors that populations are facing, providing an opportunity to assess the effect of those variables on animal’s body conditions in terms of health and nutrition [5].

## Supporting information

S1 Table List of hematological and biochemistry parameters assessed by physiographic region and area in the Greater Everglades, South Florida. Blood parameters are classified by groups (general health, nutrition, stress, inflammation/infection, dehydration, nutrition/dehydration) describing sample size per parameter (n), heteroscedasticity statistics (S p-value = Spearman p-value, ρ value), ordinary least square linear regression p-value (OLS p value), and goodness of fit values (Adj R^2^ = Adjusted R^2^). Bold numbers refer to analysis in which we obtain at least weak evidence of an effect. Outliers detected and deleted before running models in Everglades (*) and Big Cypress (**).
(XLSX)

S2 Table Multiple regression analysis models per area and region describing all the variables included per model. This table describes the model names provided in Table 3.
(XLSX)
